# Social phobic beliefs mediate the relationship between post-event processing regarding the worst socially aversive experience and fear of negative evaluation

**DOI:** 10.1007/s12144-022-02805-9

**Published:** 2022-02-07

**Authors:** Rosa J. Seinsche, Bertram Walter, Susanne Fricke, Marie K. Neudert, Raphaela I. Zehtner, Rudolf Stark, Andrea Hermann

**Affiliations:** 1 Department of Psychotherapy and Systems Neuroscience, Justus Liebig University Giessen, Otto-Behaghel-Str. 10H, Giessen, 35394 Germany; 2 Bender Institute of Neuroimaging, Justus Liebig University Giessen, Giessen, Germany; 3 Center for Mind, Brain and Behavior, Philipps University Marburg and Justus Liebig University Giessen, Giessen, Germany

**Keywords:** Post-event processing, Etiologically relevant socially aversive events, Structural equation modelling, Cognitive model of PTSD

## Abstract

The experience of socially aversive events is proposed to be a critical etiological factor in the development of social anxiety symptoms even though the experience itself is also common among healthy individuals. Rather than the event itself, accompanying factors such as maladaptive processing might be associated with higher levels of social anxiety symptoms. One-hundred-seventy-four individuals participated in this online-survey comprising questionnaires regarding social anxiety symptoms and retrospective reports concerning maladaptive processing of the worst socially aversive event. Structural equation modelling was used to analyze the hypothesized mediation of maladaptive processing and fear of negative evaluation by intrusive re-experiencing and social phobic beliefs. The positive association between retrospectively evaluated maladaptive processing after the worst socially aversive event and fear of negative evaluation was mediated by social phobic beliefs but not by intrusive re-experiencing. These results point towards the relevance of further investigating processing strategies after socially aversive events as a potential influencing factor for SAD development.

Trial registration.

The trial was registered at the German Clinical Trial Register (DRKS00021502) on June 3^rd^, 2020.

**Supplementary Information:**

The online version contains supplementary material available at 10.1007/s12144-022-02805-9.

## Introduction

Socially aversive experiences are proposed to be an important environmental risk factor for the development of Social Anxiety Disorder (SAD; Rapee & Spence, 2004; Spence & Rapee, 2016). These experiences are often linked to distorted self-images contributing to the maintenance of social anxiety symptoms by increasing the assumed threat of social situations (Clark & Wells, 1995; Hackmann et al., 1998, 2000; Rapee & Heimberg, 1997). However, the experience of socially aversive events is not limited to patients with SAD, as many healthy individuals also report having experienced such events (e.g. Bjornsson et al., 2020; Carleton et al., 2011; Erwin et al., 2006). The relationship between these socially aversive events and the development of social anxiety symptoms resembles the aetiology of Posttraumatic Stress Disorder (PTSD). Beyond that, patients with SAD also suffer from posttraumatic stress symptoms such as avoidance, hyperarousal and intrusive re-experiencing, especially in the form of distorted self-images linked to early socially aversive events (Bjornsson et al., 2020; Carleton et al., 2011; Edwards et al., 2003; Erwin et al., 2006; Hackmann et al., 2000; Mellings & Alden, 2000; Rachman et al., 2000). Both patients with SAD and PTSD further experience negative beliefs related to the aversive event increasing the assumed threat of current situations (Clark & Wells, 1995; Ehlers & Clark, 2000; Norton & Abbott, 2016; Reimer & Moscovitch, 2015; Tutus & Goldbeck, 2016). These similarities indicate potential benefits from taking PTSD models into account to investigate the relationship between the experience of socially aversive events and social anxiety symptoms (Carleton et al., 2011; Norton & Abbott, 2017; Sansen et al., 2015).

According to the cognitive model of PTSD (Ehlers & Clark, 2000), maladaptive processing strategies after traumatic events play a significant role in the development of posttraumatic stress symptoms like intrusive re-experiencing and negative event-related beliefs (Brewin et al., 2010; Ehlers & Clark, 2000; Ehlers & Steil, 1995; Turliuc et al., 2015). Strategies like rumination, thought suppression or avoidance reduce distress related to the aversive event in the short-term by distracting attention from negative feelings (Michael et al., 2007). However, in the long-term, they impede adequate processing of the event by preventing problem-solving processes and the event’s integration into long-term memory (Ehlers & Clark, 2000; Ehlers & Steil, 1995; Turliuc et al., 2015). Previous studies on the association between maladaptive processing strategies and PTSD symptoms after traumatic events have primarily been investigating by means of cross-sectional studies and retrospective evaluations of processing strategies (for an overview see Szabo et al., 2017), indicating significant associations between both constructs.

In their cognitive model of SAD, Clark and Wells (1995) propose that SAD patients also engage in maladaptive processing of current social situations. They repeatedly engage in negative reviews about the experience and especially their performance (Abbott & Rapee, 2004; Clark & Wells, 1995; McEvoy & Kingsep, 2006). This so-called ‘12’ in SAD comprises cognitive and behavioral strategies such as rumination and avoidance resembling maladaptive processing strategies of traumatic events in PTSD (Clark & Wells, 1995; Ehlers & Clark, 2000; Rachman et al., 2000). Previous studies on the relationship between post-event processing and social anxiety have shown that post-event processing of recent socially aversive events is strongly associated with social anxiety in samples of students as well as patients (Dannahy & Stopa, 2007; McEvoy & Kingsep, 2006; Mellings & Alden, 2000; Rachman et al., 2000). Similar to maladaptive processing of traumatic events in PTSD, positive associations have also been found for post-event processing of recent socially aversive events with negative social phobic beliefs and intrusive re-experiencing in response to aversive social events (Abbott & Rapee, 2004; McEvoy & Kingsep, 2006; Rachman et al., 2000). Increasing negative self-evaluations of performance, post-event processing might finally prevent distorted self-images, as well as negative self-beliefs to update (Abbott & Rapee, 2004; Mellings & Alden, 2000; Norton & Abbott, 2017). 

In sum, previous studies have demonstrated an association between etiologically relevant socially aversive events and key maintaining factors like intrusive self-images and negative self-beliefs in SAD. However, yet unclear are the processes by which these etiological and maintaining factors of SAD are linked. Previous findings from cross-sectional and longitudinal studies in PTSD show that (retrospectively evaluated) maladaptive processing strategies like rumination or avoidance are critical factors, both for the development and the maintenance of the disorder. Also in SAD, post-event processing of current aversive social situations contributes to the maintenance of anxiety symptoms. However, until now, little is known about the role of post-event processing in response to socially aversive events with potential etiological relevance. The aim of this online survey in a non-clinical student sample was to investigate retrospective reports about the intensity of post-event processing after the experience of the worst socially aversive event the participants have ever experienced. We selected the worst socially aversive event as an approximation of an etiologically relevant event because we studied a sample with varying degrees of fear of negative evaluation (FNE). More specifically, we were interested, whether individuals with higher levels of FNE retrospectively report having experienced more post-event processing after the worst socially aversive event. Fear of negative evaluation is a construct closely related to social anxiety and has shown to be suitable for analogous samples (Clark & Wells, 1995; Rapee & Heimberg, 1997; Stopa & Clark, 2001). It was also found to be associated with post-event processing in a non-clinical sample (Dannahy & Stopa, 2007). We were additionally interested in the relationship between post-event processing regarding the worst socially aversive event and intrusive re-experiencing (composed of intrusive thoughts and memories, ‚here-and-now quality’, nightmares, distress and bodily reactions while remembering) according to PTSD criteria, as well as general social phobic beliefs (composed of negative self-beliefs, expectations of social inadequacy and excessive social performance standards). Socially aversive events are associated with negative self-beliefs and intrusive re-experiencing, which in turn increase anxiety in social situations contributing to the maintenance of the disorder (e.g. Norton & Abbott, 2017). According to cognitive models of PTSD, maladaptive processing strategies of the event (resembling post-event processing in SAD) rather than the traumatic event itself play an essential role in the development of intrusive re-experiencing and negative beliefs (e.g. Ehlers & Clark, 2000). Therefore, we hypothesized that the relationship between retrospectively reported post-event processing after the worst socially aversive event and FNE is mediated by the frequency of intrusive re-experiencing regarding the same event, as well as social phobic beliefs. As retrospective estimations of post-event processing might be biased, we additionally investigated the association between emotion regulation styles and the same variables in further analyses. Results of previous studies in PTSD have indicated similarities between trait-like emotion regulation styles and event-related processing strategies (Allbaugh et al., 2016; Cann et al., 2011).

## Material and Methods

### Participants

Four-hundred-forty-seven adults (≥ 18 years) recruited from a local university’s mailing list participated in this online survey. Two-hundred-seventy participants were excluded because of early termination of the survey, and three participants were excluded because they did not report having experienced a socially aversive event, leaving a final sample of 174 participants. Participants were on average 25.07 years old (*SD* = 6.42 years, range = 18 – 56 years), and the majority of participants were female (73.0%). Sixty-nine per cent of all participants reported high school graduation as their highest educational level, 28.2% a university degree and 2.3% a secondary school leaving certificate. Prior to data collection, we determined a minimum sample size of *N* = 129 to detect significant (α = 0.05) medium-sized effects (*f *^*2*^= 0.15) for multiple regression analyses (4 predictors: FNE, post-event processing, social phobic beliefs, intrusive re-experiencing) with a power of 0.95 (G*Power, Version 3.1.9.2, Faul et al., 2007). All participants gave informed consent, and the study protocol was approved by the local ethics committee.

### Procedure

Participants were asked to fill in several questionnaires via the online tool Sosci Survey (Leiner, 2019). First, participants completed a questionnaire listing socially aversive situations and were asked to recall their worst socially aversive event. This was followed by questionnaires concerning post-event processing and posttraumatic stress symptoms, as well as characteristics of the memory (not reported in this manuscript) concerning this worst socially aversive event, as well as questionnaires regarding FNE, negative social phobic beliefs, emotion regulation strategies and depressiveness. After completing all questionnaires, participants could participate in a raffle for two 50€ vouchers.

### Questionnaires

#### Fear of negative evaluation

The intensity of FNE was assessed with the German version of the self-report questionnaire ‘Fear of Negative Evaluation Scale’ (FNES; Watson & Friend, 1969; German version: Vormbrock & Neuser, 1983). The FNES is depicting a central component of social anxiety, namely the fear to make a negative impression on other people, using 20 items rated on a 4-point scale ranging from 1 (almost never) to 4 (almost every time). Vormbrock and Neuser (1983) reported a good internal consistency for the German version, and reliability analysis in our sample revealed a Cronbach’s *α* of 0.905.

#### Social phobic beliefs

Dysfunctional social phobic beliefs were assessed using the German version of the ‘Social Attitudes Questionnaire’ (SAQ; Clark, 1995; Stangier et al., 1996). Fifty items were rated on a 7-point scale ranging from 0 (not at all) to 6 (completely) examining negative self-beliefs, expectations of social inadequacy and excessive social performance standards (e.g. german translation of “I am not acceptable.”, “My fear is obvious for other people.”, “I have to do all things right to get accepted.”). Internal consistency is reported to be acceptable to high (Mitte et al., 2007). Reliability analysis for our sample also showed good reliability (Cronbach’s *α* = 0.962).

#### Depressiveness

Depressiveness was assessed to describe the sample further and was measured with the depression scale of the German version of the ‘Depression-Anxiety-Stress-Scale-21’ (DASS-21; Lovibond & Lovibond, 1995; Nilges & Essau, 2015). Seven items were rated on a 4-point scale ranging from 0 (not at all) to 3 (very much/most of the time). The psychometric properties of the German version are reported to be good (Nilges & Essau, 2015)*.* Reliability analysis for our sample also revealed good internal consistency *(*Cronbach’s *α* = 0.909).

#### Emotion regulation strategies

The German questionnaire ‘Heidelberg Form of Emotion Regulation Strategies’ (HFERST; Izadpanah et al., 2019) was used to examine the regular usage of eight emotion regulation strategies (rumination, reappraisal, acceptance, problem solving, suppression of emotional expression, suppression of emotional experience, avoidance, social support). In this manuscript we focused on the dysfunctional strategies (rumination, avoidance, experience suppression, expressive suppression), as these strategies are described as aspects of post-event processing (see Clark & Wells, 1995). The internal consistency of the individual subscales was reported to be good (Cronbach’s *α* > 0.8, Izadpanah et al., 2019) and reliability analysis in our sample revealed acceptable to good reliability for rumination (Cronbach’s *α* = 0.882), avoidance (Cronbach’s *α* = 0.769), expressive suppression (Cronbach’s *α* = 0.716) and experience suppression (Cronbach’s *α* = 0.815). Three to four items are rated on a 5-point scale ranging from 1 (never) to 5 (always) for each subscale.

#### Socially aversive event

Participants completed a questionnaire inquiring whether they had ever experienced any of the listed socially aversive situations of various nature, such as showing poor performance in public, being ridiculed by authorities or being bullied by other children (adapted from Erwin et al., 2006). There was also an open response option to make sure that all potential situations were covered. Participants were instructed to choose the worst socially aversive situation they had ever experienced. Questionnaires concerning intrusive re-experiencing (see sect. Intrusive re-experiencing) and post-event processing (see sect. Post-event processing) refer to this worst socially aversive event. Participants were excluded if they chose an event that happened in the last four weeks because PTSD symptoms are diagnosed at the earliest four weeks after the traumatic event (Falkai et al., 2018).

#### Intrusive re-experiencing

An adapted version of the German version of the ‘Posttraumatic Diagnostic Scale’ (PTDS; Foa et al., 1997; Griesel et al., 2006) was used to investigate the frequency of the experience of posttraumatic stress symptoms related to the worst socially aversive event (see sect. Socially aversive event) in the last four weeks (e.g. intrusive re-experiencing, avoidance, hyperarousal). Besides, participants were asked if they had indicated problems (at least one of the previous section’s posttraumatic stress symptoms) related to the aversive event in the last four weeks. If they had experienced problems, it was also assessed whether these had affected participants in various areas of their lives (e.g. work, relationship with friends, eroticism). The PTDS also collects data concerning the event’s time (6 categories: less than one month ago, one to three months ago three to six months ago, six months to three years ago, three to five years ago, more than five years ago). This manuscript only focused on the intrusive re-experiencing scale composed of 5 items regarding intrusive thoughts and memories, ‚here-and-now quality’, nightmares, distress and bodily reactions while remembering. The items were rated on a 4-point scale ranging from 0 (never or only one time in the last month) to 3 (five or more times a week/nearly always), and a sum score of all five items was used. Psychometric properties of the German version of the PTDS have shown to be good (Griesel et al., 2006). Reliability analysis of the intrusive re-experiencing scale in our sample revealed a Cronbach’s *α* of 0.869.

#### Post-event processing

Post-event processing regarding the worst socially aversive event was assessed using the German version of the ‘Post-event Processing Questionnaire’ (PEPQ; Rachman et al., 2000; German revised version: Fehm et al., 2008). The PEPQ assesses post-event processing by subsuming different cognitive and emotional processing strategies after socially aversive events, e.g. thought suppression, avoidance or rumination, and negative consequences of the memory, e.g., interference with concentration and negative emotional responses. It consists of 17 items rated on a visual analogue scale from 0 (none/never/not at all) to 100 (very strong). Participants were instructed to rate the items regarding their worst socially aversive event (see sect. Socially aversive event). Reliability analysis in our sample showed good internal consistency (Cronbach’s *α* = 0.895).

## Statistical analyses

**Fig. 1:**
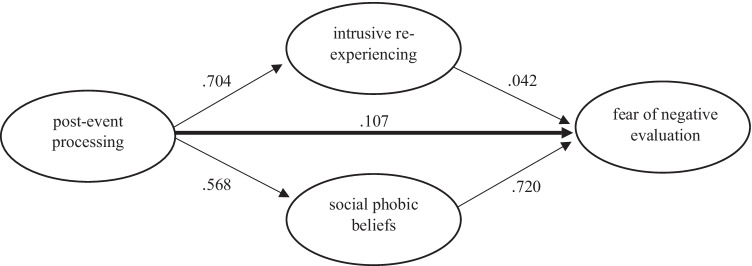
Standardized coefficients for the relationship between post-event processing (PEPQ), intrusive re-experiencing (PTDS), social phobic beliefs (SAQ) and fear of negative evaluation (FNES)

Structural equation modelling (SEM) was performed to analyze the relationship between post-event processing and social anxiety. We hypothesized a direct relationship and a mediation by intrusive re-experiencing and social phobic beliefs, respectively (see Fig. 1). Post-hoc analyses were computed with emotion regulation styles (rumination, avoidance, expressive suppression and experience suppression; instead of post-event processing) and their relationship with FNE, social phobic beliefs and intrusive re-experiencing (see Supplementary tables 1 – 5). SEM makes use of multivariate techniques to estimate the hypothesized relationship between independent and dependent variables based on the observed covariance matrix of measured variables (Beaujean, 2014) and was performed using R (Version 4.0.2; R Core Team, 2017) with the R package lavaan (Version 0.6 – 7; Rosseel, 2012).

Manifest variables in the SEM consisted of all items of the FNES (FNE), all items of the intrusive re-experiencing scale of the PTDS (intrusive re-experiencing), all items of the PEPQ (post-event processing) and all items of the SAQ (social phobic beliefs). Prior to analysis, missing data were excluded list-wise. Normality (Shapiro Wilk Test, p < 0.05), skewness and kurtosis (Mardia’s test, p < 0.05) were assessed, revealing a violation of multivariate normal distribution. Thus, we used standard maximum likelihood estimation of model parameters with robust standard errors and a Satora-Bentler scaling of test statistics. For the evaluation of overall model fit, we relied on Hooper et al., (2008), who consider a comparative fit index (CFI) > 0.95, a root mean square error (RMSEA) < 0.07, and a standardized root mean square residual (SRMR) < 0.08 as acceptable fit.

## Results

### Characteristics of the socially aversive events

The intensity of FNE and depressiveness varied largely across the sample (see Table 1), and all participants reported that they had experienced at least one socially aversive event. To further describe the socially aversive events, we report the recalled content category and timeframe of the events, which may be subject to memory bias. Categories of the worst socially aversive events reported varied between participants: 20.1% indicated that they had been rejected or excluded, 19.5% had been ridiculed by their peers, 13.2% had been criticized for their appearance, 10.9% had given a poor performance in public, 8.6% had been bullied by parents or authorities, and 8.0% chose the open response option. The remaining 19.7% of the participants reported having experienced one of the remaining categories as the worst socially aversive event (< 7% for each category). The time at which the event occurred also differed between subjects: The majority of participants reported that they remembered that the event had occurred more than five years ago (67.2%), followed by six months to 3 years ago (14.9%) and 3 to 5 years ago (13.2%). The remaining 4.6% reported that the event had occurred in the last six months. None of the participants reported that the event had happened in the previous four weeks. More than two-thirds of all participants (69.5%) reported in the PTDS (Foa et al., 1997; Griesel et al., 2006) that they had experienced posttraumatic stress symptoms related to the event in the last four weeks (e.g. intrusions, avoidance, concentration difficulties). These problems have reportedly affected participants in the following areas of their daily life: Relationships with friends (57.9%), entertainment/recreation (42.1%), education (41.3%), relationship to family members (28.9%), work (27.3%), housework (23.1%) and eroticism (23.1%). More than half of the participants (61.2%) reported that their overall life satisfaction had been affected by consequences of the event during the last four weeks.

**Table 1:** Descriptive statistics of fear of negative evaluation (FNES), social phobic beliefs (SAQ), post-event processing (PEPQ), intrusive re-experiencing (PTDS), depressiveness (DASS) and emotion regulation strategies (HFERST)

Variable	*M*	*SD*	Range
Fear of negative evaluation	52.632	11.869	23–77
Social phobic beliefs	120.402	53.643	18–271
Post-event processing	855.563	365.480	68–1700
Intrusive re-experiencing	3.718	3.641	0–15
Depressiveness	7.270	5.878	0–21
Emotion regulation strategies			
Rumination	14.977	4.251	4–20
Reappraisal	11.670	3.676	4–20
Acceptance	9.477	2.973	3–15
Problem solving	15.351	3.593	4–20
Expressive suppression	13.115	3.564	4–20
Experience suppression	10.126	3.805	4–20
Avoidance	9.828	2.817	3–15
Social support	6.345	2.659	2–10

### Mediation analysis

Correlation analyses revealed significant correlations between all four variables (see Table 2). The SEM investigated social phobic beliefs (SAQ) and intrusive re-experiencing (PTDS) as mediators between post-event processing (PEPQ) and FNE (FNES), respectively (see Fig. 1). The model displayed an acceptable fit (robust CFI = 0.688, robust RMSEA = 0.067, SRMR = 0.078). All standardized regression coefficients of latent variables on manifest variables were at least 0.160 (median = 0.640).

**Table 2:** Pearson correlations between fear of negative evaluation (FNES), social phobic beliefs (SAQ), post-event processing (PEPQ) and intrusive re-experiencing (PTDS)

Variable	Fear of negative evaluation	Social phobic beliefs	Post-event processing
Social phobic beliefs	.750		
Post-event processing	.463	.524	
Intrusive re-experiencing	.384	.419	.590

*Note.* All *p* < *.001*

We examined several paths from post-event processing to FNE (see Fig. 1 and Tables 3 and 4). The direct path showed only a small effect (*β* = 0.107). Most prominent was a path showing an effect mediated by social phobic beliefs (*β* = 0.409). Post-event processing also showed a strong association with intrusive re-experiencing (*β* = 0.704), but a relation of intrusive re-experiencing and FNE barely existed, which resulted in a mediation effect of only *β* = 0.042. Thus, we can conclude that the substantial effect of post-event processing on FNE resulted from mediation by social phobic beliefs only.

**Table 3:** Structural equation model (SEM) for the relationship between fear of negative evaluation (FNES), intrusive re-experiencing (PTDS), social phobic beliefs (SAQ), and post-event processing (PEPQ)

Path	*β*	*p*	*SE*
Fear of negative evaluation ←			
Intrusive re-experiencing	.042	.587	.094
Social phobic beliefs	.720	< .001	.142
Post-event processing	.107	.307	.177
Social phobic beliefs ←			
Post-event processing	.568	< .001	.117
Intrusive re-experiencing ←			
Post-event processing	.704	< .001	.112

*Note. β* = standardized regression coefficients

**Table 4:** Direct and indirect pathways from post-event processing (PEPQ) to fear of negative evaluation (FNES)

Pathways	*β*	*p*	*SE*
Direct pathway			
Fear of negative evaluation ← post-event processing	.107	.307	.177
Indirect pathways			
Fear of negative evaluation ← social phobic beliefs ← post-event processing	.409	< .001	.131
Fear of negative evaluation ← intrusive re-experiencing ← post-event processing	.030	.585	.092
Total effect	.546	< .001	.159

*Note. β* = standardized regression coefficients

Further correlational analyses of the relationship between dysfunctional emotion regulation styles (rumination, avoidance, expressive suppression, experience suppression), post-event processing, social phobic beliefs, intrusive re-experiencing and FNE revealed significant correlations between all variables except for expressive suppression and intrusive re-experiencing, as well as experience suppression and intrusive re-experiencing (see Supplementary table 1). Hence, separate mediation models were calculated for rumination and avoidance (see Supplementary tables 2 – 5; Bonferroni correction for multiple testing: *α* = 0.025). The relationship of both, rumination and avoidance, respectively, and FNE was mediated by social phobic beliefs but not by intrusive re-experiencing.

## Discussion

This study is a first approach to investigate maladaptive processing strategies after early socially aversive experiences (similar to PTSD). Since there are no long-term studies on this topic yet, the aim of this online survey in a student sample was to investigate retrospective reports of post-event processing after the worst socially aversive event and its association with social anxiety symptoms, similar to previous cross-sectional studies in PTSD (see Szabo et al., 2017). We expected the association of post-event processing after the worst socially aversive event and FNE to be mediated by intrusive re-experiencing of the same event as well as social phobic beliefs. As hypothesized, post-event processing after the worst socially aversive event was positively related to self-reported FNE, as well as intrusive re-experiencing and social phobic beliefs. Furthermore, the relationship between post-event processing and FNE was mediated by social phobic beliefs, while no mediation effect was found for intrusive re-experiencing. As retrospective reports regarding post-event processing can be biased, we have included current dysfunctional emotion regulation styles in the analyses to approximate a current measure of post-event processing and analyses indeed revealed similar results. However, being no substitute for prospective studies, results have to be interpreted with caution. Nevertheless, this might be a first indication of the potential role of dysfunctional processing strategies after aversive experiences, not only in PTSD but also regarding social anxiety.

Our results are in line with previous findings that the mere experience of socially aversive events is not specific for developing social anxiety symptoms, as previously demonstrated in SAD (Bjornsson et al., 2020; Erwin et al., 2006). In this heterogeneous sample in terms of FNE, nearly all (except three) participants were able to report a socially threatening event from their past. Beyond that, many participants reported that these events continued to result in various problems such as intrusive re-experiencing, avoidance or concentration difficulties that affected different areas in their lives in the last four weeks. These findings further support the relevance of investigating factors potentially contributing to the development of social anxiety after socially aversive events.

Moreover, our results show that retrospective reports of post-event processing are associated with today’s self-reported FNE, being a first indication towards an association between both constructs. These results are also in line with previous studies on post-event processing after traumatic events in PTSD, as well as post-event processing after recent socially aversive events in SAD (Brewin et al., 2010; Ehlers & Clark, 2000; Ehlers & Steil, 1995; Szabo et al., 2017; Turliuc et al., 2015). In this study, post-event processing of the worst socially aversive event was further associated with social phobic beliefs mediating the relationship between post-event processing and FNE. As in PTSD, post-event processing may lead to a biased recall of the memory by interfering with adaptive changes to the meaning of the event resulting in persistent negative self-beliefs (Brewin et al., 1996; Ehlers & Clark, 2000). However, due to our cross-sectional design, we cannot conclude the relevance of post-event processing for SAD development. We do not know whether participants with a higher level of FNE experienced increased post-event processing after the event. It is equally possible that looking back, participants with higher levels of FNE think about the event in a more negative way. In addition, the initial level of social anxiety during the time when the worst socially aversive event happened remains unknown. However, post-hoc correlational and mediation analyses revealed a significant correlation between dysfunctional emotion regulation styles and post-event processing, as well as a significant mediation effect of social phobic beliefs on the relationship between these emotion regulation styles and FNE (see Supplementary tables 1 to 5). Similarities in 14 may indicate that individuals with higher levels of trait rumination and avoidance might also have experienced more post-event processing regarding the socially aversive event. Previous studies on PTSD indicate a temporal stability of the association between trait-like emotion regulation styles and event-related processing (e.g. Allbaugh et al., 2016; Cann et al., 2011). In addition, the positive correlation between emotion regulation styles and intrusive re-experiencing after the aversive social event further points towards an association between emotion regulation strategies and PTSD symptoms in SAD patients, similar to PTSD (Frewen et al., 2012; Pugach et al., 2020; Sippel et al., 2016). At the same time, this may also indicate that the habitual use of maladaptive emotion regulation strategies might be a risk factor for post-event processing of socially aversive events and, finally, the development of social anxiety. These uncertainties demonstrate the importance of further investigating processing strategies after socially aversive events as influencing factors for the development of SAD. To rule out potential memory biases and confirm temporal relationships, longitudinal studies in children and adolescents are necessary.

Intrusive re-experiencing was found to be no significant mediator of post-event processing and FNE even though previous studies in PTSD suggest that maladaptive processing such as rumination leads to intrusive recollections of the aversive event (e.g. Ehlers & Steil, 1995; Michael et al., 2007). Posttraumatic stress symptoms after socially aversive events in SAD may differ in their form between SAD and PTSD, with a focus on intrusive distorted self-images in SAD (Carleton et al., 2011; Erwin et al., 2006; Hackmann et al., 2000), which were not explicitly assessed by the PTDS (Griesel et al., 2006). Furthermore, the PTDS assesses the frequency of intrusive re-experiencing regarding the socially aversive event in the last four weeks. The data of this study were collected during a statewide domestic quarantine due to the Covid-19 pandemic. Thus, participants probably were not confronted with many social situations at that time. However, intrusive re-experiencing regarding aversive events is typically triggered by similar stimuli (in this case, social situations) and thus might not have been as present during this time as in other months. Questionnaires concerning FNE and social phobic beliefs might not have been influenced as strongly, as they examine traits and not experiences exclusively in the last four weeks.

These are only preliminary results that must be interpreted with caution. First of all, mediation analyses in our cross-sectional design may be biased due to the lack of temporal ordering between post-event processing, intrusive re-experiencing, social phobic beliefs and FNE (Cole & Maxwell, 2003; Maxwell & Cole, 2007). The retrospective reports of the time frame, as well as further aspects (e.g. category, processing) of the experiences could also be biased. Longitudinal studies with clear temporal ordering are necessary to examine the relationship between the constructs. Moreover, the model tested in this manuscript explains only 25% of the variance of FNE. In addition to that, we derived our hypotheses based on PTSD models even though we only investigated intrusive re-experiencing and no other PTSD symptoms. We decided to examine only intrusive re-experiencing because there is already some evidence on intrusive re-experiencing in SAD and its association with post-event processing (Hackmann et al., 2000; Norton & Abbott, 2017; Rachman et al., 2000). Besides, by examining a non-clinical sample, we cannot make a statement on the clinical relevance of post-event processing after the worst socially aversive event in patients with SAD. Additionally, we did not measure social anxiety but FNE (even though the constructs are closely related; Clark & Wells, 1995; Rapee & Heimberg, 1997; Stopa & Clark, 2001).

## Conclusion

These preliminary findings are a first indication that post-event processing after the experience of socially aversive events may not only play a role in the maintenance of social anxiety but might also be an essential factor regarding etiologically relevant socially aversive events. Prospective studies are highly important to validate these results. Overlapping symptoms and similarities in the aetiology of PTSD and SAD point towards benefits from integrating both models for a better understanding of the development of SAD.

## Supplementary Information

Below is the link to the electronic supplementary material.

Supplementary file1 (DOCX 26.6 KB)

## Data Availability

The datasets are available from the corresponding author on request.
