# Supplementary material for: Social phobic beliefs mediate the relationship between post-event processing regarding the worst socially aversive experience and fear of negative evaluation
Source: Curr Psychol. 2022 Feb 7:1–10. Online ahead of print. doi: 10.1007/s12144-022-02805-9 (PMC8818836; doi:10.1007/s12144-022-02805-9)
Supplement: Supplementary file 1 — Supplementary file1 (DOCX 26.6 KB) [file 12144_2022_2805_MOESM1_ESM.docx]

**Supplementary table 1**

Pearson correlations between emotion regulation strategies (HFERST) and fear of negative evaluation (FNES), social phobic beliefs (SAQ), post-event processing (PEPQ) and intrusive re-experiencing (PTDS).

| Variable | Fear of negative evaluation | | Social phobic beliefs | | Post-event processing | | Intrusive re-experiencing | |
| --- | --- | --- | --- | --- | --- | --- | --- | --- |
|  | *r* | *p* | *r* | *p* | *r* | *p* | *r* | *p* |
| Rumination | .561 | <.001 | .430 | <.001 | .478 | <.001 | .351 | <.001 |
| Reappraisal | -.303 | <.001 | -.348 | <.001 | -.093 | .225 | -.075 | .327 |
| Acceptance | -.440 | <.001 | -.345 | <.001 | -.280 | <.001 | -.159 | .014 |
| Problem solving | -.028 | .715 | -.049 | .519 | .059 | .441 | .020 | .789 |
| Expressive suppression | .186 | .014 | .351 | <.001 | .167 | .028 | .052 | .500 |
| Experience suppression | .231 | .002 | .382 | <.001 | .211 | .005 | .136 | .073 |
| Avoidance | .460 | <.001 | .399 | <.001 | .368 | <.001 | .210 | .005 |
| Social support | -.188 | .013 | -.323 | <.001 | -.123 | .105 | -.107 | .160 |

**Supplementary table 2**

Structural equation model (SEM) for the relationship between fear of negative evaluation (FNES), intrusive re-experiencing (PTDS), social phobic beliefs (SAQ), and rumination (HFERST).

| Path | | *β* | *p* | *SE* |
| --- | --- | --- | --- | --- |
| Fear of negative evaluation ← | | | | |
|  | Intrusive re-experiencing | .007 | .901 | .098 |
|  | Social phobic beliefs | .616 | <.001 | .155 |
|  | Trait rumination | .375 | <.001 | .163 |
| Social phobic beliefs ← | | | | |
|  | Trait rumination | .480 | <.001 | .093 |
| Intrusive re-experiencing ← | | | | |
|  | Trait rumination | .431 | <.001 | .083 |

Note. β = standardized regression coefficients.

**Supplementary table 3**

Direct and indirect pathways from rumination (HFERST) to fear of negative evaluation (FNES).

| Pathways | | *β* | *p* | *SE* |
| --- | --- | --- | --- | --- |
| Direct pathway | |  |  |  |
|  | Fear of negative evaluation ← rumination | .375 | <.001 | .163 |
| Indirect pathways | |  |  |  |
|  | Fear of negative evaluation ← social phobic beliefs ← rumination | .295 | <.001 | .117 |
|  | Fear of negative evaluation ← intrusive re-experiencing ← rumination | .003 | .900 | .047 |
| Total effect | | .673 | <.001 | .208 |

Note. β = standardized regression coefficients.

**Supplementary table 4**

Structural equation model (SEM) for the relationship between fear of negative evaluation (FNES), intrusive re-experiencing (PTDS), social phobic beliefs (SAQ), and avoidance (HFERST).

| Path | | *β* | *p* | *SE* |
| --- | --- | --- | --- | --- |
| Fear of negative evaluation ← | | | | |
|  | Intrusive re-experiencing | .080 | .129 | .093 |
|  | Social phobic beliefs | .623 | <.001 | .144 |
|  | Avoidance | .310 | <.001 | .149 |
| Social phobic beliefs ← | | | | |
|  | Avoidance | .489 | <.001 | .107 |
| Intrusive re-experiencing ← | | | | |
|  | Avoidance | .293 | .001 | .092 |

Note. β = standardized regression coefficients.

**Supplementary table 5**

Direct and indirect pathways from avoidance (HFERST) to fear of negative evaluation (FNES).

| Pathways | | *β* | *p* | *SE* |
| --- | --- | --- | --- | --- |
| Direct pathway | |  |  |  |
|  | Fear of negative evaluation ← avoidance | 0.310 | <.001 | .149 |
| Indirect pathways | |  |  |  |
|  | Fear of negative evaluation ← social phobic beliefs ← avoidance | 0.305 | <.001 | .117 |
|  | Fear of negative evaluation ← intrusive re-experiencing ← avoidance | 0.023 | .124 | .028 |
| Total effect | | 0.639 | <.001 | .176 |

Note. β = standardized regression coefficients.
